# Protective effects of *Camellia japonica* flower extract against urban air pollutants

**DOI:** 10.1186/s12906-018-2405-4

**Published:** 2019-01-28

**Authors:** Minkyung Kim, Dahee Son, Seoungwoo Shin, Deokhoon Park, Sangyo Byun, Eunsun Jung

**Affiliations:** 1 Biospectrum Life Science Institute, A-1805, U-TOWER, 767, Sinsu-ro, Suji-gu, Yongin-si, Gyeonggi-do, 16827 Republic of Korea; 2 Department of Applied Biotechnology, Ajou University, San 5, Woncheon-Dong, Yeongtong-gu, Suwon, 443-749 Republic of Korea

**Keywords:** *Camellia japonica*, Anti-pollution, Urban dust, Anti-aging, Antioxidant

## Abstract

**Background:**

Exposure of skin to urban air pollutants is closely related to skin aging and inflammatory responses such as wrinkles formation, pigmentation spot, atopic dermatitis, and acne. Thus, a great deal of interest has been focused on the development of natural resources that can provide a protective effect to skin from pollutants.

**Methods:**

The antioxidative activity of *Camellia japonica* flower extract (CJFE) was evaluated by 1,2-diphenyl-2-picrylhydrazyl (DPPH) and 2,2′-azino-bis-3-ethylbenzthiazoline-6-sulphonic acid (ABTS) assay, and the inhibitory effect of CJFE by urban air pollutants-induced reactive oxygen species (ROS) production was determined in cultured normal human dermal fibroblasts (NHDFs). We additionally investigated the protective effects of CJFE against urban air pollutant using in vitro and ex vivo model.

**Results:**

CJFE with high phenolic concentration showed antioxidative activity on scavenging capacity of 1,2-diphenyl-2-picrylhydrazyl (DPPH) radicals and 2,2′-azino-bis-3-ethylbenzthiazoline-6-sulphonic acid (ABTS) radical cation in a concentration dependent manner. CJFE inhibited urban air pollutants-induced ROS generation, matrixmetalloproteinase-1 (MMP-1) production and a xenobiotic response element (XRE)-luciferase activity indicating the aryl hydrocarbon receptor (AhR) transactivation. In addition, CJFE showed an excellent protective activity against pollutants-induced deteriorating effect in ex vivo model. CJFE reduced the level of pollutants-induced malondialdehyde (MDA), lipid peroxidation marker, inhibited MMP-1 expression and increased collagen synthesis. It also reduced the cell numbers with pyknotic nuclei (mainly occurring in apoptosis) and detachment of dermo-epidermal junction (DEJ) induced by pollutants.

**Conclusions:**

Apparently, it is proposed that CJFE can be used as a protective material against pollutant-induced skin damages.

**Electronic supplementary material:**

The online version of this article (10.1186/s12906-018-2405-4) contains supplementary material, which is available to authorized users.

## Background

Air pollution has become one of the serious problems in big cities and is the leading causes of many health problems such as cardiovascular diseases, stroke as well as respiratory diseases. There are many kinds of substances that cause air pollution such as carbon dioxide, dust, ozone, noxious fumes, fine dust, etc. [1–4]. Airborne particulate matters (PMs) are classified into coarse (2.5–10 μm), fine (< 2.5 μm), and ultrafine (< 0.1 μm), which are composed of ion components such as nitrate (NO_3_^−^), ammonium (NH_4_^+^) and sulfate (SO_4_^2−^), carbon compounds and metal compounds generated by the combustion action. Polycyclic aromatic hydrocarbons (PAHs) among various pollutants constituting PMs are designated as carcinogenic substances, which are classified as toxic air pollutants. The PHAs causes changes in the DNA structure by transforming into carcinogenic substance by the action of cytochrome P450s (CYPs). The cytochrome P450s family plays a central role in xenobiotics metabolism involved in the production of procarcinogen containing benzopyrene and trihalomethanes (THMs) [5]. The aryl hydrocarbon receptor (AhR), a ligand-activated transcription factor that responds to environmental toxin, is present in the cytoplasm in a form associated with the heat shock protein Hsp90. When the AhR binds to the pollutants, it is released from the Hsp90 complex and gets activated by tyrosine kinase phosphorylation. The AhR combined pollutants enters into the nucleus and forms a complex with the AhR-nuclear transporter (ARNT). Subsequently, the AhR-ARNT complex binds to the xenobiotic responsive element (XRE) in the enhancer region of the CYP1A1 gene in order to facilitate transcription of the gene. In this process, the produced active metabolites can cause DNA mutations leading to carcinogenesis [6–8].

The human skin, the outer layer of body is constantly exposed to the environmental toxins. Increasing evidence suggests pollutants are associated with numerous adverse effect on skin, the most notable of which are inflammatory or allergic skin disease and skin aging [9–13]. Oxidative stress and AhR activity are an important pathophysiological mechanism of pollutant-induced skin damage. Air pollution increases ROS production, mitochondrial damage and skin aging. In addition, exposure to substances such as PAHs causes skin aging by activating MMP-1 through AhR pathway [14–17]. AhR activation mediates PM-induced up-regulation of COX_2_ expression and PGE_2_ production in the skin barrier [18]. Until date, not enough research has been done on natural materials that have the potential to prevent the process of skin damage induced by air pollutants. Thus, in the present study, we have concentrated on the protective effect of the natural materials against the harmful effects caused by urban air pollutants.

*Camellia japonica* flowers are native to Korea, China, and Japan, and many horticultural varieties are being developed worldwide. Numerous biological activities of *C. japonica* such as antioxidant, anti-bacterial, anti-allergic, and anti-inflammatory activities have been reported [19–22]. However, the anti-pollution effects of *C. japonica* have not yet been elucidated. In this study, we investigated the protective effects of *C. japonica* in normal human dermal fibroblasts (NHDFs) and skin explants model against many harmful influences caused by urban air pollutants.

## Methods

### Materials

Urban dust 1649b was purchased from National Institute of Standard and Technology (NIST) (Gaithersburg, MD, USA). Diphenyl-1-picrylhydrazyl (DPPH), 2,2′-azino-bis(3-ethylbenzothiazoline-6-sulphonic acid (ABTS), 3-(4,5-dimethyl-2-thiazolyl)-2,5-diphenyltetrazolium bromide (MTT), TRIzol agent and gallic acid were supplied by Sigma (St Louis, MO, USA). 2′,7′-dichlorofluorescin diacetate (DCFH-DA) was purchased from Thermo-Fisher (Waltham, MA, USA).

### Preparation of *C. japonica* flower extract

*Camellia japonica* was harvested in Jeju island of South Korea, and was identified by Dr. Yong-Hwan Jung in Jeju Biodiversity Research Institute (JejuTechnopark, South Korea), where a voucher specimen was stored (BS00962). The raw extracts were desiccated by using hot air drier at 50 °C overnight were pulverized. The dried flower of *Camellia japonica* (100 g) was extracted with 70% (*v*/v) ethanol at room temperature overnight. After the process of filtration, the solvent was removed by a rotary vacuum evaporator (Heidolph, Schwabach, Germany) and freeze-drying was proceeded. The dried powder of *Camellia japonica* flower (7.93 g) were dissolved in DMSO for the experiments.

### Determination of antioxidant activity

The DPPH scavenging ability was measured as previously described [23]. The reaction mixture containing concentrations of CJFE and DPPH solution was incubated for 30 min at room temperature. The antioxidant activity was monitored by measuring the absorbance at 517 nm using a Gen 5™ UV-Vis spectrophotometer (BioTek, Winooski, VT, USA).

The ABTS radical scavenging assay was performed according to the previously described method with some modifications [24]. After reacting 7.4 mM ABTS with 2.6 mM potassium persulfate, the working solution was incubated for 24 h at room temperature in the dark. The reaction mixture obtained by mixing CJFE and working solution were incubated for 30 min at room temperature in the dark. Subsequently, the absorbance was measured at 732 nm using a Gen 5™ UV-Vis spectrophotometer (BioTek, Winooski, VT, USA).

### Determination of Total phenolic content

The Folin-Ciocalteu method was used to measure the amount of total polyphenols [25]. Folin-Ciocalteu phenol reagent and aqueous sodium carbonate solution (20%) was mixed. The reaction mixture was mixed well and incubated at room temperature for 30 min. The absorbance was measured at 725 nm using a Gen 5™ UV-Vis spectrophotometer (BioTek, Winooski, VT, USA). The total polyphenolic content was determined using gallic acid as a standard curve.

### Cell culture and toxicity assay

Normal human dermal fibroblasts (NHDFs) were purchased from ATCC (Manassa, VA, USA). Normal human dermal fibroblasts (NHDFs) maintained in Dulbecco’s Modified Eagle’s Medium (DMEM) with 10% fetal bovine serum (FBS), penicillin (100 U/mL), and streptomycin (100 μg/mL) at 37 °C in a humidified atmosphere containing 5% CO_2_ in the air. Cultured NHDFs were obtained from ATCC (Manassas, VA, USA) and the passages 3 to 5 were used for the experiments.

Cytotoxicity was determined by the MTT assay. NHDFs were incubated with CJFE for 72 h. Subsequent to addition of MTT solution (1 mg/mL in PBS) into each well, the cells were incubated for 2 h at 37 °C in 5% CO_2_. DMSO was added after removing the MTT solution and the absorbance was measured at 570 nm using a spectrophotometer.

### ROS production

The level of intracellular ROS was measured by the 2′7-dichlorofluorescein diacetate (DCFH-DA) in NHDFs. The cells were treated with CJFE for 2 h prior to treatment with urban dust. After incubation for 72 h, the cells were treated with 50 μM DCFH-DA for 30 min and ROS production was analyzed using an Infinite® F200 PRO (Tecan, AG, Mannedorf, Switzerland). ROS generation was expressed as a percentage of the fluorescence of untreated control.

### XRE luciferase activity assay

Cignal XRE Reporter luciferase activity assay was conducted in NHDFs using XRE reporter assay kit (QIAGEN, Maryland, USA). Cells were incubated in 6-well plate for 24 h. After transfection for 4 h, CJFE was pretreated for 2 h, and urban dust was added to the cells. After incubation for 24 h at 37 °C in 5% CO_2,_ luciferase activity analysis was conducted using Luciferase Assay System (Promega, Madison, USA).

### MMP-1 inhibition assay

NHDFs were seeded at 3 × 10^4^ /well in 12-well plates and incubated overnight at 37 °C. CJFE was pretreated into plates for 2 h and urban dust was added. After incubation for 72 h, the production of MMP-1 (Human pro-MMP-1 Immunoassay; R&D systems, Minneapolis, MN, USA) was determined using a commercial ELISA kit.

### Quantitative real-time PCR analysis

Total RNA samples were extracted using TRIzol protocol and cDNA synthesis was performed using amfiRivert cDNA synthesis platinum master mix (GenDEPOT) according to the standard method. The PCR reactions were carried out on 7300 real time PCR system (Applied Biosystems) in 96-well plates using SYBR green master mix (Applied Biosystems). MMP-1 (PPH00120B), CYP1A1 (PPH01271F) and GAPDH (PPH00150F) for qPCR primer assay were purchased from QIAGEN and used in the experiments. Relative quantification of gene expression was determined by comparative cycle time Ct (ΔΔCt) method.

### Ex-vivo analysis

The 19 skin explants of an average diameter of 11 mm (±1 mm) on an abdomen-plasty were derived from a Caucasian person in their 50’s were prepared after removing the subcutaneous fat from the skin. The explants were tested in BIO-EC’s explants culture medium at 37 °C in a humid and 5%-CO_2_ atmosphere for 5 days. On 4th day, the explants were treated with the pollution mixture of heavy metals and hydrocarbons for 24 h. The tested explants were collected and separated into two parts. One part was fixed in buffered formalin and the other was frozen at − 80 °C. After 24 h of fixation, the samples were dehydrated and embedded in paraffin using Leica TP1010 dehydration automat (Leica, Rueil-Malmaison, France) 7 μm thick sections of paraffin-embedded samples were cut for light microscopy and mounted on the Superfrost® plus silanized glass slides. To observe general morphological change, the paraffinized sections were stained with Masson’s trichrome, Goldner variant. Collagen 1 immunostaining were performed on frozen sections with a rabbit anti-collagen I polyclonal antibody (Monosan, Ref.: PS047) diluted at 1:400 in PBS, BSA 0.3% and Tween 20 (0.05%) for 1 h at room temperature using Vectastain Kit Vector amplifier system avidin/biotin, and revealed by AF488 (Lifetechnologies, Ref. A11008). Nuclei was post-stained with propidium iodide. MMP-1 immunostaining was performed on paraffinized sections with a mouse anti-MMP-1 monoclonal antibody (R&D Systems, clone 36,665) diluted at 1:25 in PBS, BSA 0.3% and Tween 20 (0.05%) for 1 h at room temperature using Vectastain Kit Vector amplifier system avidin/biotin, and observed by Vector® VIP peroxidase substrate (Vector Labs, Ref. SK-4600). All immunostaining processes were performed using an automated slide-processing system (Dako, AutostainerPlus) and observed by microscopical observation (Leica DMLB or Olympus BX43 microscope). The MDA assay was examined with an enhanced method of the TBARs (ThioBarbituric Acid Reagents) assay. The level of MDA was measured in spectro-fluorimetry (excitation 515 nm, emission 550 nm) using a microplate reader (Tecan Infinite M200 Pro).

### Statistical analysis

All data are expressed as mean ± SD of three independent experiments. *P < 0.05, **P < 0.01 vs. pollutant-untreated control. #*P* < 0.05, ##*P* < 0.01 vs. pollutant-treated control.

## Results

### Inhibitory effect of CJFE on urban pollutants induced ROS production

**Fig. 1:**
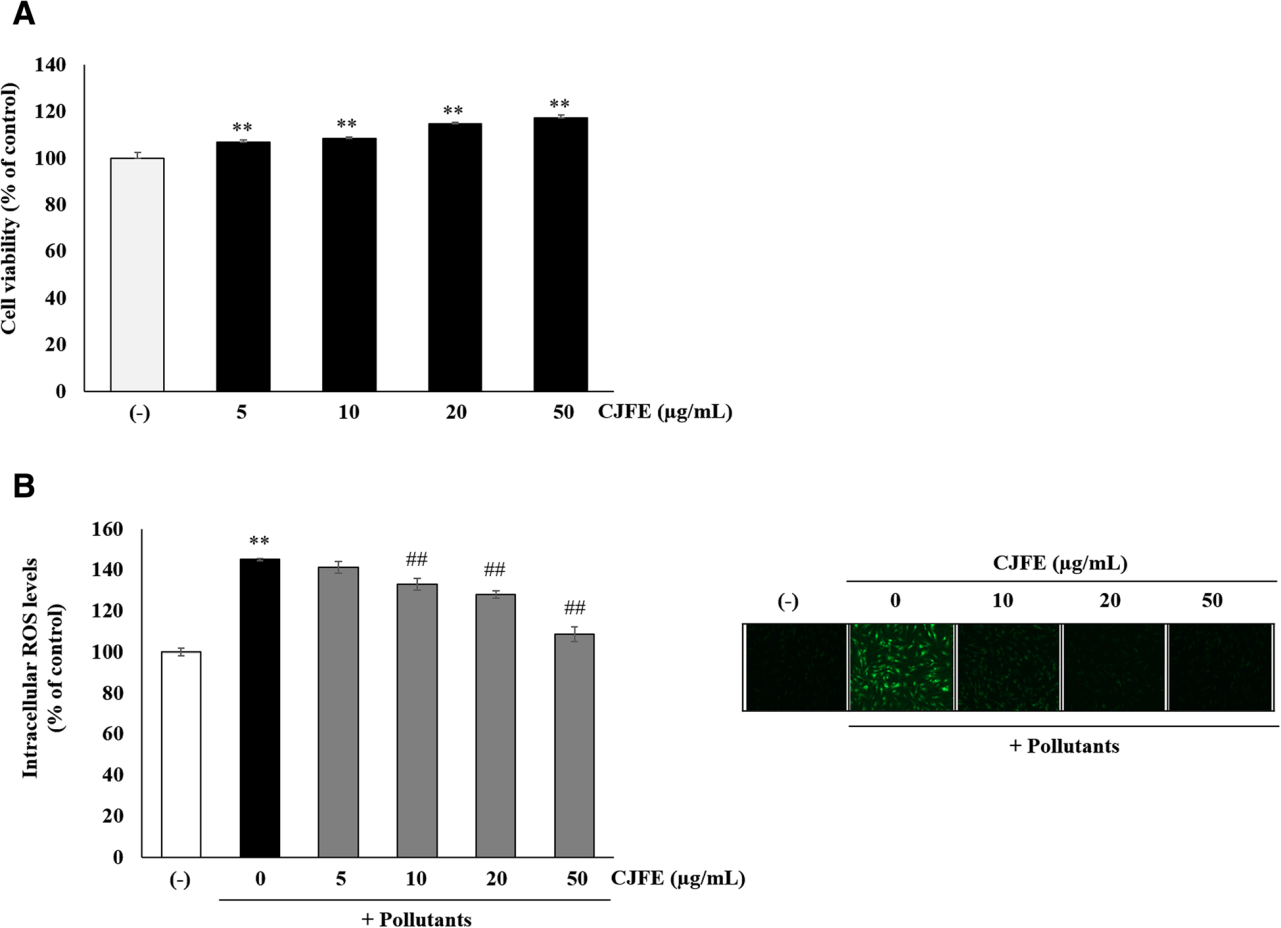
Cell viability (**a**) and intracellular ROS levels (**b**) in normal human dermal fibroblasts (NHDFs). Cell toxicity test in NHDFs was assayed by MTT assay. Intracellular ROS levels were determined by measuring the oxidative conversion of DFH-DA to the DCF. Fibroblasts were harvested and incubated with 50 μM DCFH-DA at 37 °C for 30 min in the dark. The fluorescence intensity was monitored by an Infinite® F200 PRO. The results are mean ± standard deviation (SD) (*n* = 3). *P < 0.05, **P < 0.01 vs. pollutant-untreated control. #*P* < 0.05, ##*P* < 0.01 vs. pollutant-treated control

We determined cell viability using MTT assay and scavenging ability of CJFE investigating intracellular ROS levels in NHDFs in the presence of CJFE (Fig. 1). CJFE was not cytotoxic up to 50 μg/mL in NHDFs.

In this study, we used a standard reference material (SRM) called as urban dust (1649b) from NIST and employed it in our experiment. The urban dust (1649b) is the most similar component to atmospheric particulate matters, and consists of PAHs, chlorinated pesticides, polychlorinated biphenyl (PCB) congeners, inorganic constituents and nitro-substituted PAHs (nitro-PAHs). CJFE treatment in NHDFs led to a significant reduction in intracellular ROS production increased by urban dust. These results indicate that CJFE acts as a safe and powerful radical scavenging agent in the intracellular environment of polarity.

### Free radical scavenging activity and total phenolic content of CJFE

**Fig. 2:**
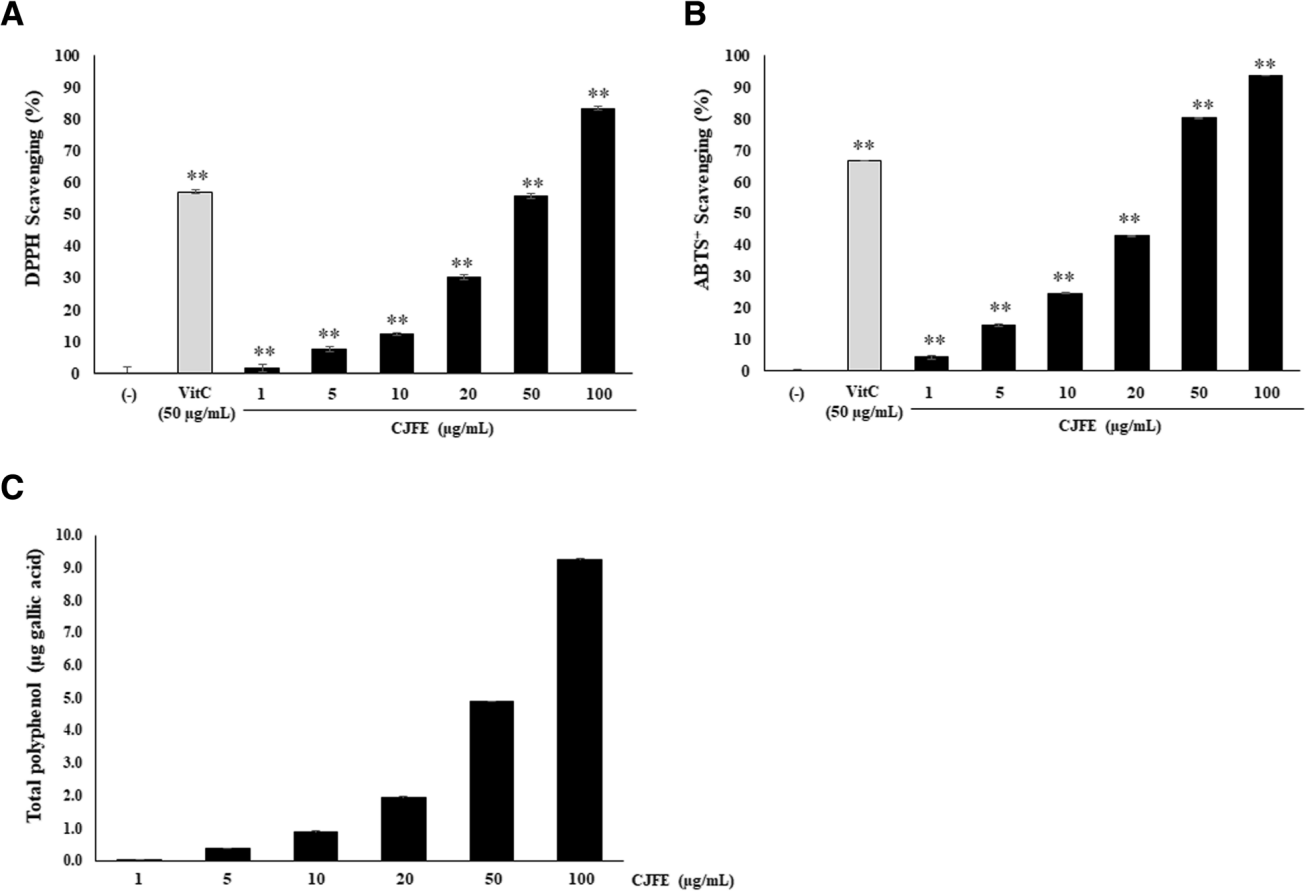
The antioxidant effects of *Camellia japonica* flower extract. The antioxidant capacity and total phenolic content of CJFE were evaluated by DPPH (**a**) ABTS (**b**) and phenolic content assay (**c**). The results are mean ± standard deviation (SD) (n = 3). *P < 0.05, **P < 0.01 vs. pollutant-untreated control. #*P* < 0.05, ##*P* < 0.01 vs. pollutant-treated control

To evaluate the antioxidant activity of CJFE, DPPH, ABTS^+^ scavenging activity and total phenolic content assay were performed (Fig. 2). CJFE at a concentration of 50 μg/mL showed better ABTS radical cation scavenging ability than Vitamin C (50 μg/mL). Total phenolic content of CJFE were measured using gallic acid as the standard by the Folin-Ciocalteu method. CJFE showed high phenolic content (97.7 μg GA/mg). The results indicated that CJFE with high phenolic content significantly exhibited strong antioxidant properties in a dose-dependent manner as neutralizing free radicals by donating a hydrogen atom or an electron.

### Inhibitory effect of CJFE on urban pollutants induced AhR-signaling pathway

**Fig. 3:**
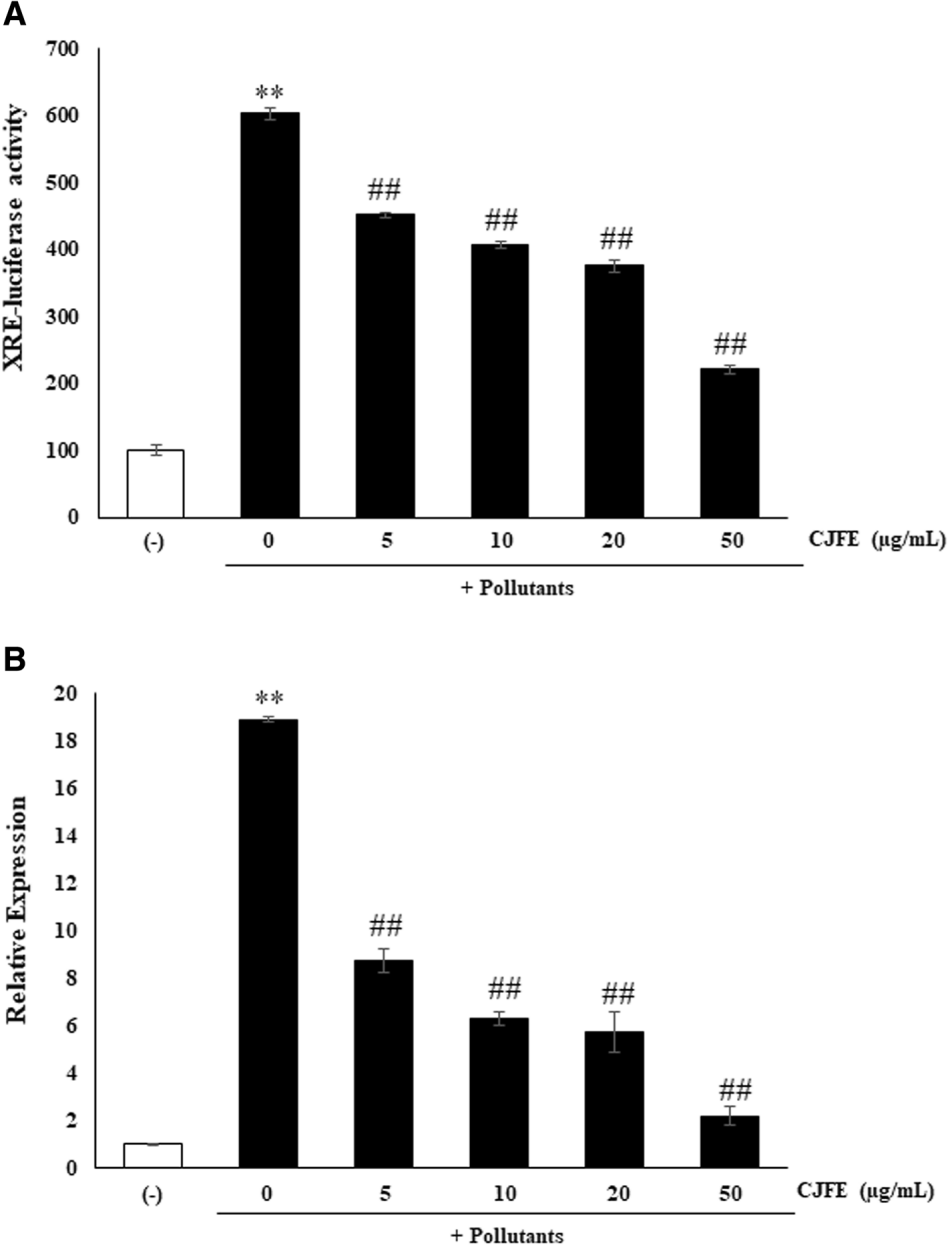
Inhibitory effect of CJFE on the AhR signaling pathway induced by urban air pollutants. The inhibitory effect of CJFE on the AhR signaling pathway was determined by measuring the XRE-luciferase activity (**a**). The level of CYP1A1 expression in NHDFs was evaluated by quantitative RT-PCR assay (**b**). The results are mean ± standard deviation (SD) (n = 3). *P < 0.05, **P < 0.01 vs. pollutant-untreated control. #*P* < 0.05, ##*P* < 0.01 vs. pollutant-treated control

Inhibition effect of CJFE on activated AhR-signaling pathway by urban pollutants was evaluated by in vitro luciferase activity and the level of CYP1A1 mRNA expression in NHDFs. The cells were incubated with various concentrations of CJFE (5, 10, 20, and 50 μg /mL) and then exposed to the urban air pollutants (50 μg /cm^2^ per plate area). As evident in Fig. 3, it was confirmed that CJFE decreased pollutants-induced luciferase activity in a dose-dependent manner, and CJFE significantly inhibited XRE-luciferase activity at a concentration of 50 μg/mL. Furthermore, CJFE decreased the level of CYP1A1 mRNA expression increased by urban pollutants. The results indicated that urban air pollutants activate the AhR-signaling pathway, and CJFE decreases the activated signaling pathway.

### Inhibitory effect of CJFE on urban pollutants induced MMP-1

**Fig. 4:**
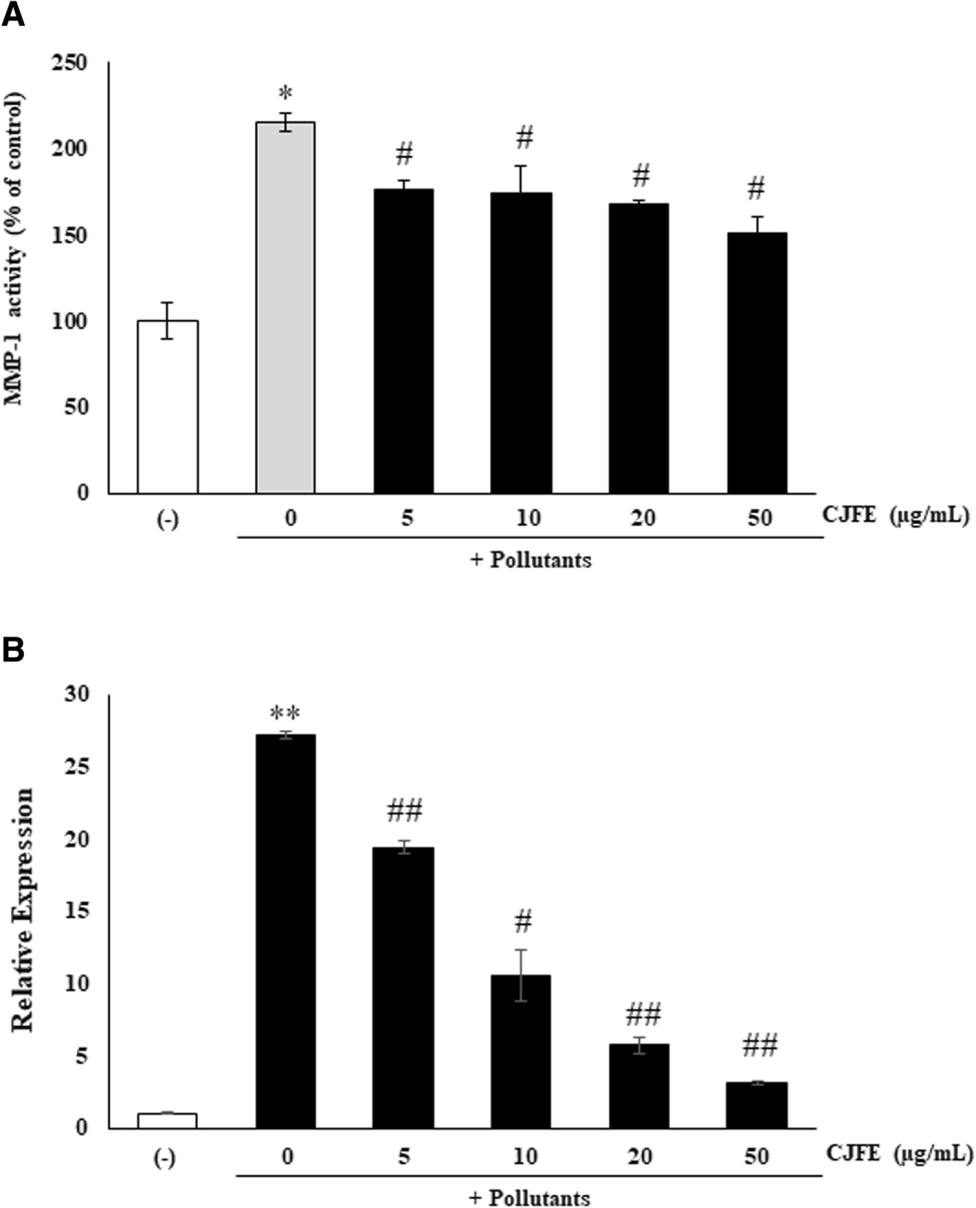
Anti-pollution effects of CJFE assessed by MMP-1 inhibition (**a**) and quantitative RT-PCR results of urban dust-induced MMP-1 expression in NHDFs (**b**). The results are mean ± standard deviation (SD) (n = 3). *P < 0.05, **P < 0.01 vs. pollutant-untreated control. #*P* < 0.05, ##*P* < 0.01 vs. pollutant-treated control

To further confirm the anti-pollution effects of CJFE, we measured mRNA levels of MMP-1 in NHDFs using quantitative RT-PCR. CJFE decreased the expression level of MMP-1 mRNA induced urban pollutants in a dose-dependent manner. In addition, CJFE reduced MMP-1 activity induced by exposure to the urban air pollutants in the cells (Fig. 4).

### Protective effect of CJFE on human living skin explants

**Fig. 5:**
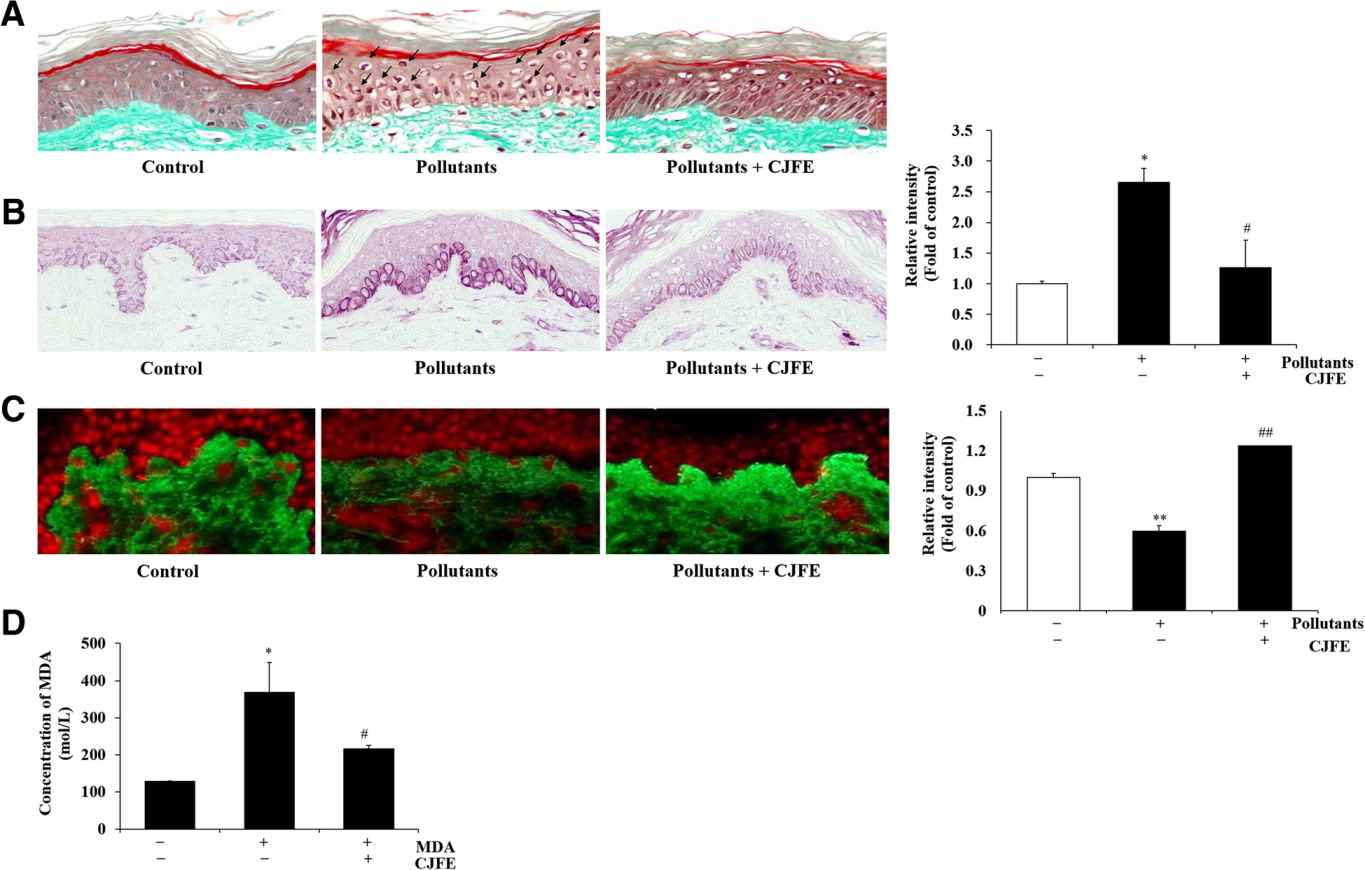
The anti-aging activity of CJFE on human living skin explants exposed to pollutants. CJFE exhibited a protective effect on pyknotic nuclei condensed by pollutants as a result of morphological analysis (**a**). The expression of MMP-1 increased by pollutants was markedly decreased by treatment with CJFE (**b**), increase of Collagen I (**c**) and decrease in the level of MDA compared to the control (**d**). The results are mean ± standard deviation (SD) (n = 3). *P < 0.05, **P < 0.01 vs. pollutant-untreated control. #*P* < 0.05, ##*P* < 0.01 vs. pollutant-treated control

To further investigate if CJFE could protect the skin from the pollutants, we assessed anti-aging effects of CJFE in skin explants (Fig. 5). The experiment was performed for 5 days by local treatment of skin explants (2 mg/cm^2^) with CJFE. In the group treated with pollutants, a mixture of heavy metals and hydrocarbons were treated on the 4th day and then were sampled after 24 h. In general morphology, CJFE inhibited the alterations in skin morphology induced by pollutants. In a pollutant-treated group, a high number of pyknotic nuclei cells as a cell death marker, and increased detachment of the dermal-epidermal junction (DEJ) and decreased dermal matrix were observed. However, CJFE treatment of pollutant-exposed skin sample reduced the number of pyknotic nuclei cells and the detachment of DEJ and increased the decreased dermal matrix. In addition, CJFE showed inhibitory effect with the increase in pollutant-induced MMP-1 expression and increased the collagen I expression decreased by pollutants. It also reduced pollutant-induced malondialdehyde (MDA) production as a lipid peroxidation marker. These results are consistent with the results of in vitro effect of CJFE in NHDFs.

## Discussion

Skin aging is characterized by loss of collagen, a major component of extracellular matrix (ECM) [26, 27]. Reduced collagen level in the dermis results from decreased collagen synthesis and increased MMPs activity responsible for the degradation of collagen and other proteins in ECM. Several studies reported that environmental pollutants such as polycyclic aromatic hydrocarbons (PAHs), oxides, ozone, and cigarette smoke accelerate extrinsic skin aging via AhR/ROS/MMPs or ROS/MMPs signaling pathway. Cigarette smoke is reported to increase the MMPs via activation of the AhR pathway and ROS generation, and smokers have more facial wrinkles than non-smokers [28–35]. The AhR / ARNT complex in the nucleus binds to a xenobiotic responsive element (XRE) sequence, resulting in induction of the cytochrome P450 enzyme family, CYP1A1. [6–8]. CYP1A1 convert PAHs to quinones which induce ROS generation [36]. Pollutants directly induce the generation of ROS such as superoxide (O2•–) and hydroxyl radical (•OH) with subsequent cellular oxidative stress. Pollutant-induced ROS generation mediates adverse effects on the skin like DNA damage, and induction of proinflammatory cytokine and MMPs [37, 38]. In this study, we confirmed that urban-dust increased ROS generation in NHDFs, and treatment with CJFE at the non-toxic concentration led to a decrease in the intracellular ROS levels in a dependent manner (Fig. 1). Additionally, we observed free radical scavenging effects of CJFE at low concentration. The ABTS^+^ scavenging effect was much better when compared with the effect of vitamin C (50 μg/mL). Furthermore, CJFE inhibited pollutant-induced XRE-luciferase activity and its downstream regulated gene CYP1A1. The induction of the MMP-1 was downregulated by CJFE in a concentration-dependent manner. Taken together, CJFE inhibited the urban dust-induced MMP-1 expression via suppression of ROS generation and AhR signaling pathway in fibroblasts. There are many reports on the effect of pollutants on the epidermis, but the effects of pollutants on the dermal layer with emphasis on penetration are not well elucidated. Therefore, it is very imperative to study the effect of pollutants on dermal as well as epidermal layers of the skin.

The protective effects of CJFE on human living skin explants exposed to pollutants were analyzed by measuring four parameters, general morphology, and production of collagen I, MMP-1 and malondialdehyde (MDA). Based on the morphological analysis, CJFE exhibited a protective effect on skin against pollutants as a result of reducing pyknotic nuclei, preventing DEJ detachment and increasing ECM density. DEJ is a part of the human skin between the epidermis and the dermis, which support the epidermis and become partially functions as a skin barrier as it forms an adhesive. A reduction in the level of DEJ proteins occurs during skin aging that leads to a loss of mechanical support to the epidermis and reduction in metabolic communication between the cellular layers [39]. UV radiation is reported to induce DEJ damage by activation MMPs and plasmin [40]. Activation of MMPs by pollutants could be the possible reason behind DEJ detachment in our model; however, the mechanism of DEJ damage induced by pollutants needs to be further investigated. The increase in MMP-1 expression caused by pollutants was more prominent in the basal layer of epidermis near DEJ area than dermis. MMP-1 expression induced by pollutants in keratinocytes is confirmed based on the results of in vitro experiment (Additional file 1: Figure S1). CJFE significantly inhibited MMP-1 expression and an attenuated decrease in collagen I induced by pollutants. Furthermore, the amount of MDA was significantly reduced by CJFE. Therefore, we can substantiate the results of the anti-aging activity test of CJFE against the pollutant-stimulated human living skin explants with the results of in vitro experiments.

## Conclusions

In conclusion, the skin exposed to air pollution can undergo acceleration in the skin-aging process as well as inflammation. Our results suggest that *Camellia japonica* flower extract (CJFE) has protective effects against the mechanism of skin aging activated by urban air pollutants (Additional file 2: Figure S2). Consequently, it is proposed that CJFE can be used as possible natural-based ingredient to prevent air pollutants-induced skin aging.

## Additional files

Additional file 1: **Figure S1.** Inhibitory effect of CJFE on urban pollutant induced XRE luciferase activity in Human keratinocyte immortal cell line (HaCaT) (TIF 12 kb)

Additional file 2: **Figure S2.** A representative figure on the protective effect of CJFE against urban air pollutants (TIF 412 kb)

## Abbreviations

ABTS: 2,2′-azinobis-3-ethylbenzthiazoline-6-sulphonic acid
AhR: Aryl hydrocarbon receptor
ARNT: AhR nuclear translocator
CJFE: *Camellia japonica* flower extract
DEJ: Dermo epidermal junction
DPPH: 1,2-diphenyl-2-picrylhydrazyl
GAPDH: Glyceraldehyde 3-phosphate dehydrogenase
MDA: Malondialdehyde
MMP-1: Matrix metallopeptidase 1
PAHs: Polycyclic aromatic hydrocarbons
XRE: Xenobiotic responsive element
