# Supplementary figures and images for: Protective effects of Camellia japonica flower extract against urban air pollutants
Source: BMC Complement Altern Med. 2019 Jan 28;19:30. doi: 10.1186/s12906-018-2405-4 (PMC6350298; doi:10.1186/s12906-018-2405-4)

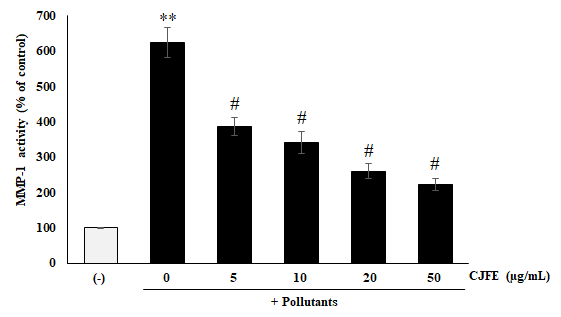

Supplement: Supplementary file 1 — Figure S1. Inhibitory effect of CJFE on urban pollutant induced XRE luciferase activity in Human keratinocyte immortal cell line (HaCaT) (TIF 12 kb) [file 12906_2018_2405_MOESM1_ESM.tif]

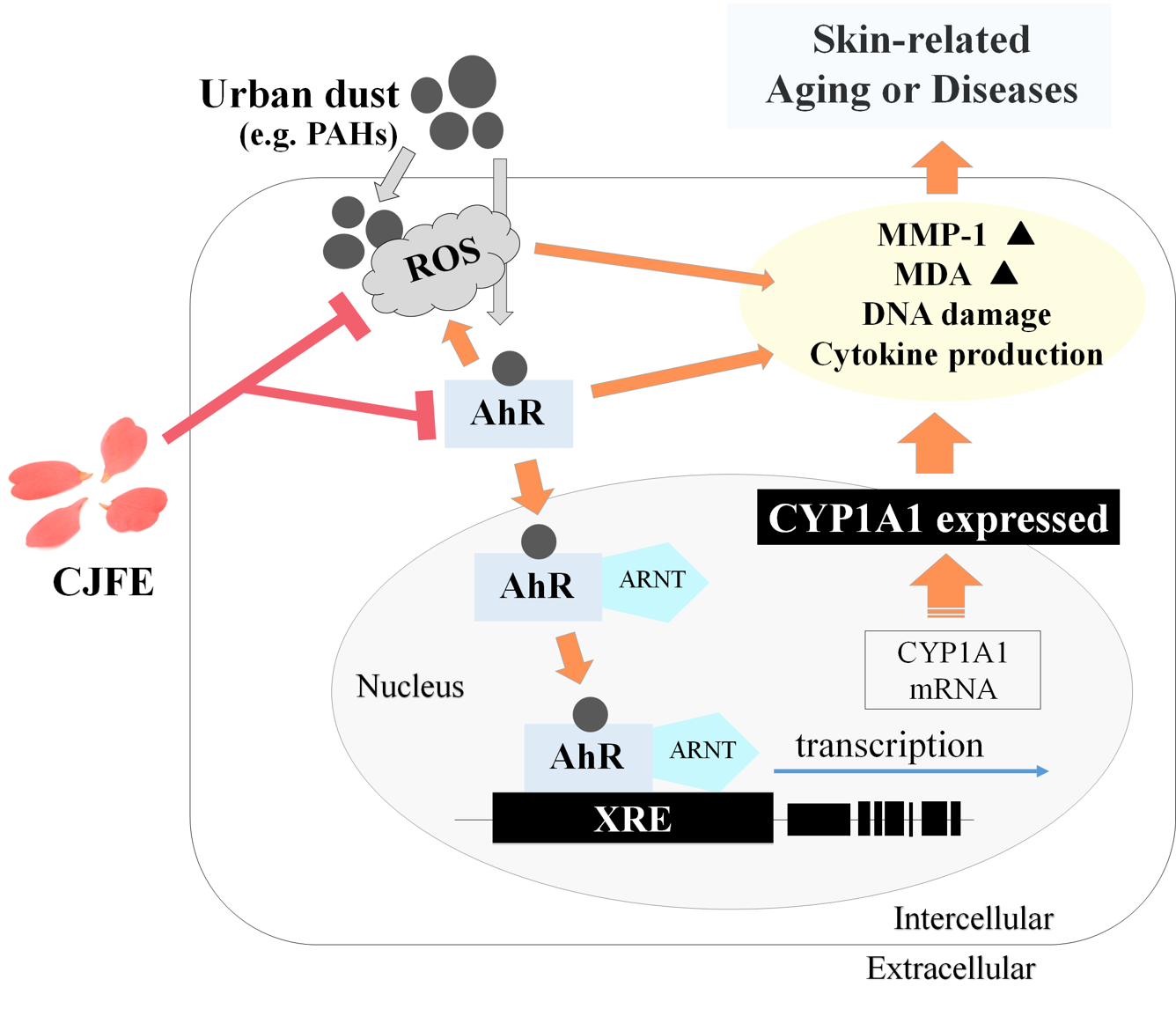

Supplement: Supplementary file 2 — Figure S2. A representative figure on the protective effect of CJFE against urban air pollutants (TIF 412 kb) [file 12906_2018_2405_MOESM2_ESM.tif]
